# Utilizing imbalanced electronic health records to predict acute kidney injury by ensemble learning and time series model

**DOI:** 10.1186/s12911-020-01245-4

**Published:** 2020-09-21

**Authors:** Yuan Wang, Yake Wei, Hao Yang, Jingwei Li, Yubo Zhou, Qin Wu

**Affiliations:** 1 College of Artificial Intelligence, Tianjin University of Science and Technology, TianJin, 300222 China; 2 Population and Precision Health Care (Tianjin), Ltd, TianJin, China; 3 Center for Cyber Security, University of Electronic Science and Technology of China, ChengDu, China; 4 Department of Critical Care Medicine, West China Hospital, Sichuan University, ChengDu, China; 5 State Key Laboratory of Information Security, Institute of Information Engineering, Chinese Academy of Sciences, Beijing, China

**Keywords:** Acute kidney injury (AKI), Prediction, Ensemble learning, ETSM, Drug combination

## Abstract

**Background:**

Acute Kidney Injury (AKI) is a shared complication among Intensive Care Unit (ICU), marked by high cost, high morbidity and high mortality. As the early prediction of AKI is critical for patients’ outcomes and data mining is such a powerful prediction tool, many AKI prediction models based on machine learning methods have been proposed. Our motivation is inspired by the fact that the incidence of AKI is a changing temporal sequence affected by the joint action of patients’ daily drug combinations and their physiological indexes. However, most existing models have not considered such a temporal correlation. Besides, due to great challenges caused by sparse, high-dimensional and highly imbalanced clinical data, it is hard to achieve ideal performance.

**Methods:**

We develop a fast, simple and less-costly model based on an ensemble learning algorithm, named Ensemble Time Series Model (ETSM). Besides benefiting from vital signs and laboratory results as explicit indicators, ETSM explores the effect of drug combinations as possible implicit indicators for the AKI prediction. The model transforms temporal medication information into a multidimensional vector to consider and measure drug cumulative effects that may cause AKI.

**Results:**

We compare ETSM with state-of-the-art models on ICUC and MIMIC III datasets. On the basis of the experimental results, our model obtains satisfactory performance (ICUC: AUC 24 hours ahead: 0.81, 48 hours ahead: 0.78; MIMIC III: AUC 24 hours ahead: 0.95, 48 hours ahead: 0.95). Meanwhile, we compare the effects of different sampling and feature generation methods on the model performance. In the ablation study, we validate that medication information improves model performance (24 hours ahead: AUC increased from 0.74 to 0.81). We also find that the model’s performance is closely related to the balanced level of the derivation dataset. The optimal ratio of major class size to minor class size for the model is found for AKI prediction.

**Conclusions:**

ETSM is an effective method for the early prediction of AKI. The model verifies that AKI incidence is related to the clinical medication. In comparison with other prediction methods, ETSM provides comparable performance results and better interpretability.

## Background

Acute Kidney Injury (AKI), a sudden loss of kidney functions, is a shared complication in the Intensive Care Unit (ICU) patients [1]. The incidence of AKI usually causes a significant drain on medical resources and increases patients’ morbidity and mortality [2]. It is noteworthy that timely detection and management can effectively reverse patients’ conditions. Therefore, the early prediction of AKI helps physicians give patients timely medical interventions and is critical for improving patients’ outcomes.

The application of machine learning methods in clinical endpoint prediction works has greatly boomed in recent years [3–6]. AKI prediction is in the spotlight and usually modeled as a classification problem in the machine learning field. The methods currently adopted by researchers can be divided into statistical machine learning methods, such as Gradient Boosting Machine [7], Random Forest [8] and Logistic Regression [9], and deep learning methods, such as Recurrent Neural Network and Multilayer Perceptron[10, 11]. Mostly, these models mainly use raw data directly as their predictors. For example, Flechet et al. [12] used patient demographics, past medical history, vital signs, and laboratory values as the input features. However, these prediction models are usually limited by the following defects:

- Failure to offer satisfactory prediction performance. Deep learning models with relatively better results pay the high cost on calculation and real-time updates.
- Failure to consider the temporal correlation of electronic health data and the influence of drug combination.

In our previous work[13], we have developed a method to extract features from the medication information and this method is helpful for AKI prediction. This paper extends that work with the following significant improvements. 1) More comprehensive experiments are conducted on more datasets and new findings are reported. 2) The interpretability of our model is analyzed.

The incidence of AKI is a changing temporal sequence affected by the joint action of patients’ daily drug combination and their physiological index. Therefore, the time series modeling method is reasonable and essential in AKI prediction because it enables instant correlation of electronic health data and is more medically interpretable. Moreover, multiple factors, such as patients with no diagnoses, no treatments, or missing records, cause electronic health data sparsity. Besides, owing to the specificity of clinical data, AKI patients of the whole cohort are often in the minority. Such an imbalanced dataset also makes the prediction difficult.

To solve the above problems, we propose an Ensemble Time Series Model (ETSM) for AKI early prediction. First, to utilize the temporal correlation of data, we creatively design a fast and straightforward time-series model. Then, to cope with the sparsity of data, the XGBoost algorithm that we used has a strong tolerance to missing values. In comparison with other prediction methods, ETSM provides comparable performance results and better interpretability.

To mitigate the class imbalance problem, we implement and compare the performance of random undersampling, random oversampling and cost-sensitive XGBoost. Based on overall performance, we finally select the random undersampling technique and implement ETSM on two datasets. According to the experimental results, ETSM offers satisfactory early prediction performance in both internal validation and external validation (ICUC: AUC 24 hours ahead: 0.81, 48 hours ahead: 0.78; MIMIC III: AUC 24 hours ahead: 0.95, 48 hours ahead: 0.95). Medication information is verified to improve the model performance (ICUC: 24 hours ahead: AUC increased from 0.74 to 0.81). Such performance improvement also shows that medication information is related to AKI incidence. To further improve model performance, the missing values are filled with the adjacent timestamp. If a sample’s values of a specific feature are entirely missing, they would be filled with the median. We also find the optimal ratio of AKI patients and non-AKI patients when training the model. Through comparing with other feature generation methods, it is proved that our approach can not only obtain comparable prediction performance but also offer guidance for medical intervention.

The paper is arranged as follows. Details about the feature generation process and the ETSM for AKI prediction are presented in Method. Statistical information of data and experimental results are shown in Results. Our discovery is discussed in Discussion and we summarized this paper in Conclusion.

## Methods

### Problem formulation

In this study, we formulate the early prediction of AKI as a classification task. Classified samples are patients, who are represented by a series of values on the dataset. These values contain an ID number, vital signs, laboratory results and medication information. Each patient has a unique ID number, which helps to identify him/her. Vital signs and laboratory results record the value of the patient’s physiological index and medication information record drugs used by the patient. For convenience, we use *d*_*i*_ to represent a kind of drug. These values reflect the patient’s physical condition and treatment received during hospitalization and are organized in chronological order.

AKI patients, the samples of key research, are defined as the positive class. Correspondingly, we define non-AKI patients as the negative class. Then the early prediction of AKI is to determine whether a sample is positive or negative with features generated from values that represent patients.

### Feature generation

Considering the sparsity and temporal correlation of clinical data, features for each patient used in this study are generated from sequential vital signs, laboratory results and medication information through a creative method. We model this information into two groups of features, *explicit indicator group* and *implicit indicator group*.

#### **Definition 1**

(Explicit indicator) Explicit indicator is the feature generated from vital signs and laboratory results value.

Vital signs and laboratory results reflect patients’ physical condition directly. Naturally, models can infer patients’ condition through fluctuations in such indexes. These parts of data can be transformed into the model’s features without complicated steps, helping to predict AKI. Therefore, we define features generated from vital signs and laboratory results as explicit indicator group.

Explicit indicator group includes two parts, ICU admission day’s data and predictive point’s data. Patients’ vital signs and laboratory results values at these two days are directly used.

**Fig. 1:**
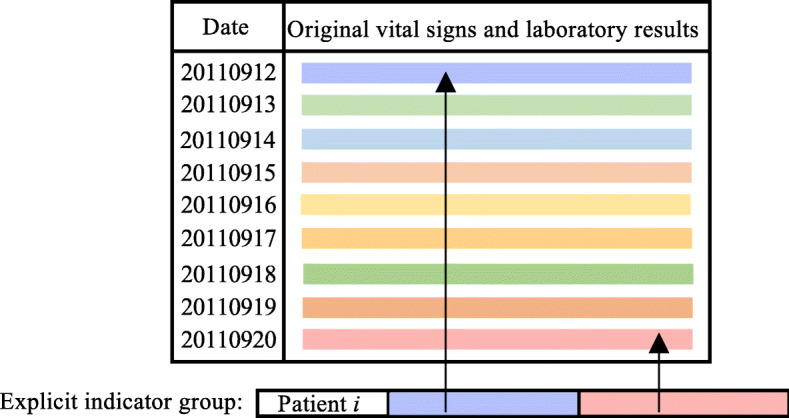
Feature geeration process of explicit indicator group. Take patient *i* as an example, the duration of ICU stay is from 20110912 to 20110920. Each color block stands for a series of vital signs and laboratory results value on a certain. This figure was generated by PowerPoint 2019

For example, in Fig 1, we extract vital signs and laboratory results of patient *i* at the ICU admission day, “20110912”, and predictive point, “20110920” as the explicit indicator group of input feature.

#### **Definition 2**

(Implicit indicator) Implicit indicator is the feature generated from medication information.

The medication patients used does not directly reflect the patients’ condition. For example, we can not figure out the patient’s heart rate from his/her heart rate through medication information. The relationship between patients’ condition and medication need to dig. Therefore, features generated from medication are defined as the implicit indicator group. Moreover, recent research has proved that some drug combinations worsen patients’ conditions [14]. Information hiding in the medication records, especially drug combination, needs further digging. Since the side effects of many drug combinations are unclear in clinical so far, the physician is hard to consider the effect of all drug combinations.

**Fig. 2:**
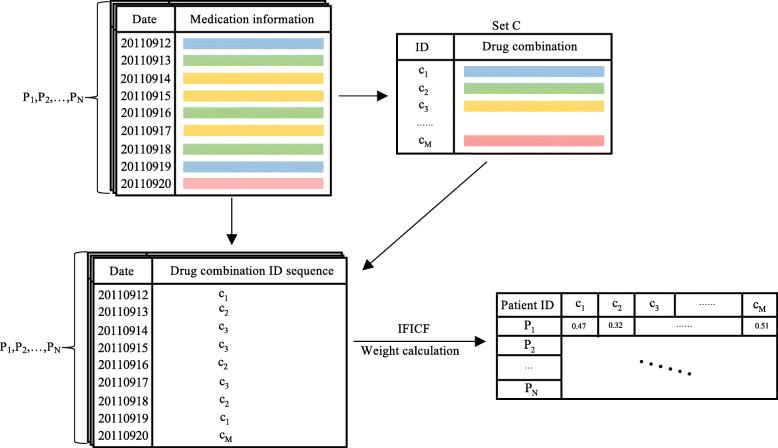
Feature generation process of implicit indicator group. *P*_*i*_ is the patient ID number. Each color block stands for a series of drugs taken on a certain day. This figure was generated by PowerPoint 2019

Medication information of patients is taken as time series. Beginning from sparse and high-dimensional data, we adopt a method, aiming to dig valid information. Figure 2 shows the complete process.

Firstly, daily drug intake of patients is recorded in a table, in which drug combination is considered as a unit. A drug combination is the combination of drugs taken by a patient on a certain day. For patient *i*, he/she used drugs *d*_1_,*d*_2_,…,*d*_*j*_ on day *t*. Therefore we define a drug combination of *c*_*k*_={*d*_1_,*d*_2_,…,*d*_*j*_}. Collecting drug combinations from the whole cohort, we obtain the drug combination set *C*={*c*_1_,*c*_2_,…,*c*_*M*_}, which is unordered and distinct. Then the medication information of patient *i* can metamorphose into sequence *s*_*i*_={*c*_1_,*c*_2_,…,*c*_*N*_}. Patients may take the same drugs on different dates and different patients perhaps take the same drugs. Drug combination set *C* is classified as the implicit indicator group. Each element in *C* represents a drug combination and an indicator of the group.

After obtaining the drug combination ID sequence for each patient, the next step is to calculate a proper weight for each indicator. The count of each indicator is related to the length of the sequence, so the importance cannot be objectively measured. Indicator Frequency and Inverse Cohort Frequency (IFICF) is a numerical statistic that indicates how paramount a drug combination *c*_*k*_ is to a sequence *s*_*i*_ in the cohort. The IFICF value increases proportionally to the number of times a drug combination *c*_*k*_ appears in the sequence *s*_*i*_ and is offset by the number of sequences in corpus *P* that contain the drug combination *c*_*k*_, which assists in adjusting for the case that some drug combinations appear more widely and frequently than others.

IFICF is the product of two statistics, Indicator Frequency (IF) and Inverse Corpus Frequency (ICF). IF is the frequency that a drug combination occurs in the drug combination ID sequence of a patient, calculated by Eq(1), where $f_{c_{k},s_{i}}$ represents the number of times drug combination *c*_*k*_ occurs in sequence *s*_*i*_ and $\sum \limits _{c\epsilon C}f_{c,s_{i}}$ means the total number of drug combinations in sequence *s*_*i*_. ICF is a measure of how important a drug combination is over the corpus, calculated by Eq(2), where *N* represents the number of patients in corpus *P* and $n_{c_{k}}$ means the number of drug combination ID sequences having drug combination *c*_*k*_.

1 $$ IF(c_{k},s_{i})=\frac{f_{c_{k},s_{i}}}{\sum \limits_{c\epsilon C}f_{c,s_{i}}}   $$

2 $$ ICF(c_{k},P)=log\left(\frac{N}{1+n_{c_{k}}}\right),n_{c_{k}}=\left| \{s \epsilon P, where\ c_{k} \epsilon s \} \right|   $$

3 $$ IFICF=IF\times ICF   $$

The patient’s feature representation of the implicit indicator group is generated after obtaining the drug combination ID sequence for each patient and calculating each indicator’s proper weight through the IFICF method. The IFICF method is derived from TFIDF (Term Frequency and Inverse Document Frequency), reflecting the idea of transforming time series modeling into text modeling. Since the definition of term and document is quite different from that in natural language processing, we redefine IFICF to help readers, especially those who are unfamiliar with this method, to understand our ideas.

In the feature generation process, we generate the explicit indicator group from vital signs and laboratory results. This group represents the patient’s physiological indexes at ICU admission day and the predictive point. It reflects changes in the patient’s physical condition during this period. These changes are closely related to AKI incidence. Then we first creatively propose a method used to generate the implicit indicator group from medication information. This group represents how vital a drug combination is to a patient and the whole cohort. Considering we generate drug combinations as features, the correlation between drug combination and AKI can be searched through this method.

### Prediction model

Since we formulate the early prediction of AKI as a classification task, the sparse and high-dimensional clinical data makes it challenging. Meanwhile, we hope our model can learn the effect of drug combination on AKI, aiming to offer help to clinical data analysis.

Due to the above reasons, we consider XGBoost as the classification model. Among the machine learning methods used in practice, XGBoost, a scalable machine learning system for tree boosting, runs faster when producing large amounts of data and skillfully handling sparse data. According to Chen et al. [15], XGBoost can run more than ten times faster than existing popular algorithms on a single machine and handle billions of samples in distributed or memory-limited settings on the same datasets comparing other tree algorithms, such as Gradient Boosting Machines. In recent years, the XGBoost algorithm has been proved that produced high predictive accuracy on classification problems and performed brilliantly in many other fields [16, 17]. The experiment of Nguyen et al. [16] exhibited that the XGBoost algorithm offered the highest accuracy level among XGBoost, Support Vector Machine (SVM), Random Forest (RF) and k-Nearest Neighbor. These features just fit the needs for countering sparse and high-dimensional clinical data when predicting AKI.

### Imbalanced dataset

In this study, the early prediction of AKI is formulated as a binary classification task. Model performance has a great relationship with the balance of the dataset. The imbalanced dataset often has a bad effect on the model’s prediction [18]. However, in the case of the clinical dataset, the negative class is much more than the positive one. To address this issue, we implement and compare random undersampling, random oversampling and cost-sensitive XGBoost. Finally, we choose to undersample the imbalanced dataset on the derivation set, aiming to better train the model. In subsequent experiments, we find it improves experimental results in the case of the highly imbalanced dataset.

### Ensemble time series model

**Fig. 3:**
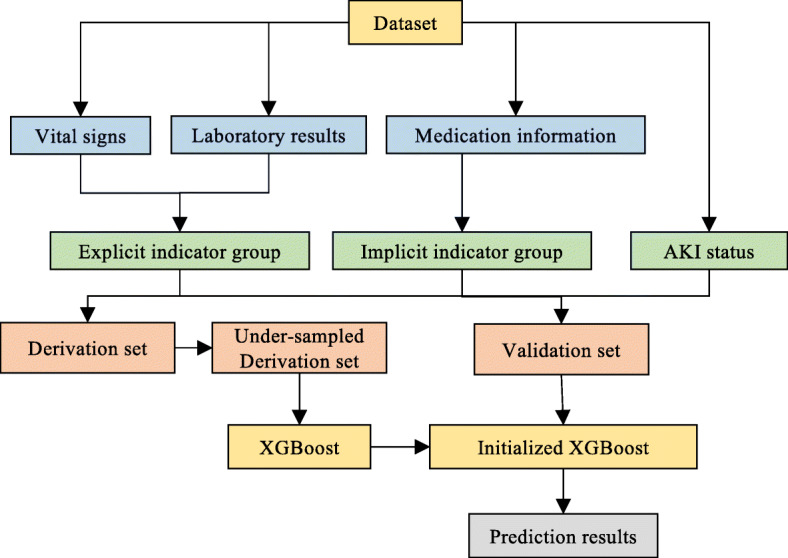
Framework of ETSM. This figure was generated by PowerPoint 2019

Figure 3 exhibits the framework of ETSM. Patients’ vital signs, laboratory results and medication records are organized by date in our dataset. It is common to set the predictive point before one or two days at clinical endpoint prediction. Therefore, our prediction model is intended to predict AKI 24 hours or 48 hours ahead before its onset, which enables patients to receive timely treatment.

Firstly, we extract vital signs, laboratory results, medication information and patients’ AKI status from the dataset. Secondly, we generate an explicit indicator group from vital signs and laboratory results. Then we generate an implicit indicator group from medication information. Thirdly, we divide samples into the derivation set and validation set. Fourthly, the XGBoost model is trained with an undersampled derivation set. Last, we examination the model performance on the validation set and get the prediction results.

This study was approved by the institutional review boards at West China hospital and was granted a waiver of informed consent (2019-S-361). Since this work is a retrospective study, the ethics committee did not require each patient to sign the informed consent.

## Results

In this section, we first introduce statistical information of two datasets and experimental settings. Secondly, we compare the performance of random undersampling, random oversampling and cost-sensitive XGBoost. Thirdly, we empirically evaluate the effectiveness of ETSM for the early prediction of AKI on the datasets. Fourthly, we discuss the data imbalance problem and the effectiveness of feature generation design.

### Data description

To verify our model’s effectiveness, we use two ICU patient collections observed in different hospitals and different countries for evaluation. We first validate our model on a local dataset, ICUC (ICU data in China), supported by Westchina critical care information system. To further verify the scalability of our model, we experiment on the external dataset. A publicly available, large-scale ICU dataset, MIMIC III (Medical Information Mart for Intensive Care)[19], is used for data extraction and model validation.

In this study, AKI is defined as serum creatinine increases by 0.3 mg/dl (26.5 *μ*mol/l) or more in 48 hours or a rise to at least 1.5-fold from baseline within seven days in light of Kidney Disease Improving Global Outcomes (KDIGO) classification [20]. Table 1 shows the summary characteristics of the study samples. It should be noted that in the MIMIC III dataset, we treat a unique icustay ID as a sample.

**Table 1:** Study sample characteristics of ICUC and MIMIC III

**Subject**	**ICUC**	**MIMIC III**
Original samples	13053	52152
AKI samples	2035	29344
Timing of AKI onset	Avg 3.92	Avg 2.00
Timing of AKI onset	Max 30	Max 7
Timing of AKI onset	Min 0	Min 0
Vital signs and laboratory results	101	38
Distinct drug	75	3235
Drug combination	5154	3085
Most widely used drug in samples	91.49%	68.82%
Patients with insufficient information	1401	5559
Samples used to predict AKI 24 hours ahead	11501	46593
Samples used to predict AKI 48 hours ahead	10921	30217

The number of samples in the negative class is usually far more than the positive class is universal trouble of the clinical dataset. ICUC is also imbalanced, and the negative class is almost 6.7 times the positive class. The original cohort contains 13053 patients, among which 2035 patients have developed AKI, accounting for about 16%. According to statistical results, samples developed AKI on average about the fourth day after ICU admission. 101 vital signs and laboratory results were recorded. 75 distinct drugs were included. On the ICUC, 5154 drug combinations are ever used by samples. The most widely used drug in samples covered 91.49% samples.

MIMIC III is much more balanced than ICUC. The original cohort contains 52152 patients, among which 29344 patients have developed AKI. On average, patients of MIMIC III suffered AKI on the second day after ICU admission. 38 vital signs and laboratory results were recorded. 3235 distinct drugs were included. On the MIMIC III, 3085 drug combinations are ever used by samples. The most widely used drug in samples covered 68.82% samples.

**Fig. 4:**
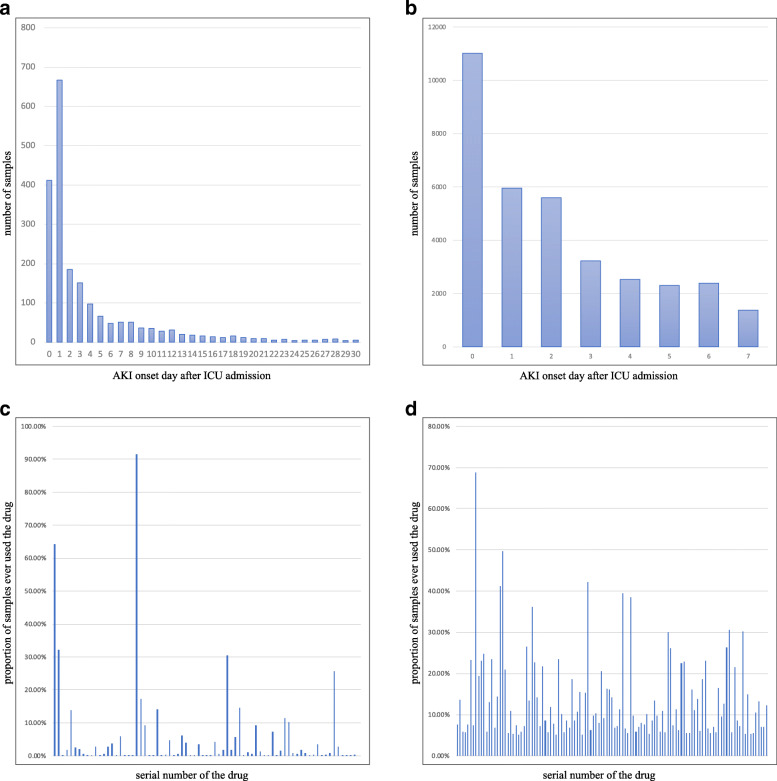
Study sample characteristics of ICUC and MIMIC III. **(a)** ICUC-timing of AKI onset **(b)** MIMIC III-timing of AKI onset **(c)** ICUC-drugs popularity **(d)** MIMIC III-drugs popularity. These figure were generated by Excel 2019

In Fig. 4(a) and (b) separately show the distribution of the timing of AKI onsets. Most patients developed symptoms within the first three days of ICU admission on the two datasets. (c) and (d) show the use range of different drugs on the two datasets.

In the raw data, there are some invalid samples with no vital signs, laboratory results, or medication information and thus need to be removed. Moreover, patients with a length of hospitalization are shorter than 24 hours or 48 hours and are also removed in the corresponding experiment. Table 1 shows the number of removed samples.

We did not use demographics features in this paper, such as age, sex. On the one hand, ICUC is a retrospective dataset. These demographics features are with a lot of missing value and correctness cannot be guaranteed. On the other hand, in the practical situation, especially in developing countries, this information is not always available.

### Experimental settings

In this study, we compare our model with single classifiers, embracing Naive Bayes and k-Nearest Neighbor, and ensemble classifiers, including AdaBoost and Random Forest, on the dataset.

The selection of the evaluation metric is critical. The area under the receiver operating curve (AUC), sensitivity, F1-score and Average Precision (AP) are selected to estimate model performance.

We calculate AUC by Eq(4).

4 $$  AUC=\frac{\sum_{{ins_{i}\epsilon positiveclass}}rank_{ins_{i}}-\frac{M \times(M+1))}{2}}{M \times N}  $$

where *M* is the number of positive class, and *N* is the number of negative class. $rank_{ins_{i}}$ represents the possibility rank of sample *ins*_*i*_ in the positive class. AUC indicates classifiers’ ability to distinguish both positive and negative classes. Even in the condition of the highly imbalanced dataset, it can still put forward sensible evaluation.

We calculate sensitivity by Eq(5).

5 $$  Sensitivity=\frac{TurePositive}{Positive Class}  $$

It is the ratio of correctly-classified positive samples to all positive samples. Sensitivity indicates the capacity of classifiers for classifying positive class unerringly.

We calculate F1-score by Eq(6).

6 $$  F1-score=\frac{2*Precision*Sensitivity}{Precision+Sensitivity}  $$

7 $$  Precision=\frac{TurePositive}{TurePositive+FalsePositive}  $$

Precision represents the ability of the model to make positive predictions correctly. It is ideal for both the precision and sensitivity score to be high. However, these two scores are contradictory and cannot be double-high. Therefore, F1-score, the harmonic mean of precision and sensitivity, is an appropriate choice.

AP is the area under the PR curve. Taking the sensitivity score as the horizontal axis and the precision score as the vertical axis, the PR curve can be drawn. This area can represent the overall performance of the model on precision and sensitivity.

For model development, the dataset was randomly divided into a derivation set (60%) used to train the model and a validation set (40%) to test the accuracy of the model, using the stratified sampling method. All experiments were repeated ten times. To mitigate the imbalanced dataset problem, we adopt a strategy that undersamples the negative class on the derivation set to produce a proper ratio of the number between positive and negative classes when fitting model. We do not make this adjustment when validating model performance.

### Comparison of imbalanced learning techniques

The research has shown that for some basic classifiers, the balanced data set provides better performance than the imbalanced data set [21]. Sampling methods are dedicated to constructing a more balanced datasets to improve model performance. Random oversampling and random undersampling are classic sampling techniques. Random oversampling technique balances data by randomly duplicating samples of the minority class, while random undersampling technique randomly drops samples of the majority class. In addition, XGBoost also provides a solution. Cost-sensitive XGBoost can offer better performance on binary classification problems with a severe class imbalance. We implement three techniques and compare model performance on the ICUC dataset. Table 2 shows the model performance of random undersampling, random oversampling and cost-sensitive XGBoost.

**Table 2:** Performance of imbalanced learning techniques on ICUC

	AUC		Sensitivity		F1-socre		AP	
	24h	48h	24h	48h	24h	48h	24h	48h
Random Undersample	0.81	0.78	0.75	0.68	0.58	0.44	0.59	0.41
Random Oversample	0.78	0.69	0.64	0.43	0.62	0.44	0.66	0.46
Cost-sensitive XGBoost	0.78	0.70	0.64	0.45	0.61	0.46	0.67	0.47

According to the result, the random undersampling technique has better AUC and sensitivity scores but also has worse F1-score and AP. However, in the longer predictive period, the random undersampling technique expands its advantages and reduced the disadvantage. Additionally, the random undersampling technique not only reduces running time and but also helps prevent overfitting [22]. Therefore, we select the random undersampling technique in the follow-up experiment.

### Performance comparisons

Tables 3 and 4 respectively exhibit the performance of prediction models in the experiment of predicting AKI incidence 24 hours and 48 hours ahead on ICUC and MIMIC III. For model development, we use the grid search to estimate the best parameters for baseline models. k-Nearest Neighbor has 6 neighbors. Random Forest has 140 estimators, and AdaBoost has 200 estimators. XGBoost has 144 estimators. The max depth of XGBoost is 8, and min child weight is 5.

**Table 3:** Performance of prediction models on ICUC

Model	AUC		Sensitivity		F1-score		AP	
	24h	48h	24h	48h	24h	48h	24h	48h
ETSM	0.81	0.78	0.75	0.68	0.58	0.44	0.59	0.41
AdaBoost	0.78	0.75	0.66	0.62	0.54	0.41	0.60	0.41
Random Forest	0.73	0.75	0.51	0.59	0.54	0.44	0.60	0.40
Naive Bayes	0.53	0.52	0.09	0.05	0.15	0.07	0.60	0.41
k-Nearest Neighbor	0.63	0.62	0.37	0.30	0.36	0.31	0.59	0.41

**Table 4:** Performance of prediction models on MIMIC III

Model	AUC		Sensitivity		F1-score		AP	
	24h	48h	24h	48h	24h	48h	24h	48h
ETSM	0.95	0.95	0.95	0.98	0.96	0.98	0.98	0.98
AdaBoost	0.89	0.93	0.93	0.97	0.93	0.96	0.98	0.98
Random Forest	0.78	0.78	0.91	0.97	0.86	0.91	0.93	0.97
Naive Bayes	0.67	0.65	0.61	0.66	0.68	0.73	0.82	0.86
k-Nearest Neighbor	0.72	0.82	0.64	0.83	0.76	0.88	0.83	0.93

In conformity to the experimental results, ETSM comes into the possession of the best result on forecasting AKI incidence both 24 hours (ICUC: AUC 0.81; MIMIC III: AUC 0.95) and 48 hours ahead (ICUC: AUC 0.78; MIMIC III: AUC 0.95). It offers brilliant outcomes at the sensitivity both 24 hours (ICUC: 0.75; MIMIC III: 0.95) and 48 hours ahead(ICUC: 0.68; MIMIC III: 0.98). ETSM also outperforms on F1-score (ICUC: 24 hours ahead: 0.58, 48 hours ahead: 0.44; MIMIC III: 24 hours ahead: 0.96, 48 hours ahead: 0.98) and provides competitive performance on AP compared to other models. In general, ensemble learning algorithms perform better than base algorithms, especially in terms of sensitivity. The experimental results demonstrate that base classifiers are not up to such complex classification problems with high-dimensional input features.

An ablation study where we exclude the implicit indicator group on ICUC is carried out so as to investigate the contribution of medication information to the model. Model with the explicit indicator group as its only input feature is named ETSM-ex. The performance of ETSM-ex in the predictive experiment is exhibited in Tables 5 and 6. It is transparent that ETSM has better performance than ETSM-ex. This advantage is particularly prominent in the experiment of predicting AKI 24 hours ahead (AUC ETSM: 0.81, ETSM-ex: 0.74). Furthermore, the incorporation of medication information brings considerable growth to the sensitivity score, F1-score and AP increase (24 hours: sensitivity increased from 0.63 to 0.75, F1-score increased from 0.47 to 0.58, AP increased from 0.47 to 0.59). In closing, the incorporation of medication information heightens the model performance.

**Table 5:** Performance of derived ETSM on ICUC in the experiment of predicting AKI 24 hours ahead

Model	AUC	Sensitivity	F1-score	AP
	(95% CI)	(95% CI)	(95% CI)	(95% CI)
ETSM	0.810 ±0.002	0.746 ±0.003	0.577 ±0.003	0.594 ±0.004
ETSM-ex	0.737 ±0.002*	0.629 ±0.004*	0.470 ±0.002*	0.470 ±0.003*
ETSM-bool	0.759 ±0.002*	0.654 ±0.005*	0.512 ±0.003*	0.530 ±0.004*
ETSM-times	0.803 ±0.002*	0.726 ±0.003*	0.579 ±0.003	0.647 ±0.003

Note: CI = confident interval

^*^indicates ETSM significantly outperforms the baseline with p <0.01 using Student t-test

**Table 6:** Performance of derived ETSM on ICUC in the experiment of predicting AKI 48 hours ahead

Model	AUC	Sensitivity	F1-score	AP
	(95% CI)	(95% CI)	(95% CI) 7 (95% CI)	
ETSM	0.776 ±0.002	0.683 ±0.004	0.437 ±0.003	0.406 ±0.004
ETSM-ex	0.775 ±0.003	0.684 ±0.006	0.434 ±0.003	0.396 ±0.005*
ETSM-bool	0.786 ±0.003	0.702 ±0.005	0.453 ±0.003	0.434 ±0.006
ETSM-times	0.806 ±0.002	0.739 ±0.006	0.476 ±0.003	0.476 ±0.005

Note: CI = confident interval

^*^indicates ETSM significantly outperforms the baseline with p <0.01 using Student t-test

### Performance with different model initialization

As mentioned above, the imbalanced dataset has a significant impact on the performance of the model. To mitigate this effect, we construct a series of derivation sets with different positive and negative proportions to train the model. Then compare their performance on the original imbalanced dataset. The proportion of positive to negative class ranges from 10:1 to 1:10.

**Fig. 5:**
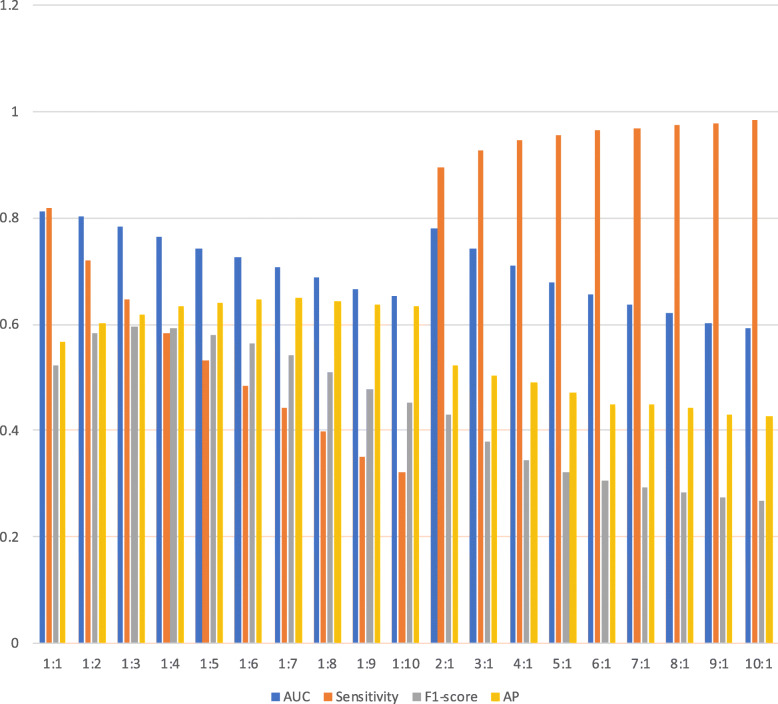
Performance of ETSM with different initialization on ICUC in the experiment of predicting AKI 24 hours ahead. This figure was generated by Excel 2019

Figure 5 demonstrates differently trained models’ performance in the predictive experiment. As can be seen in Fig 5, the AUC and sensitivity score gradually decrease along with the increase in positive class accounted for. However, the two scores have opposite development trends along with the increase in negative class accounted for. The AUC score gradually declines while the sensitivity score gradually rises. Both of F1-score and AP show the same trend of first rising and then falling.

Comprehensively compare the performance on different derivation sets, 1:1 or 1:2 ratios between positive and negative classes lead to better experimental results.

### Feature generation methods comparisons

In this part, we test another two feature generation methods and implement them with the original XGBoost model on ICUC, aiming to explore the effect of different feature generation methods on the prediction results.

#### Compared methods

In previous research, patients’ vital signs and laboratory results are generated in the same way as mentioned in our method, but medication information was often considered as distinct drugs and directly generated as features. We keep the explicit indicator group unchanged and use two other implicit indicator group generation methods to compare their performance.

- **Bool-Drug Method:** In this method, each dimension of the implicit indicator group stands for a kind of drug. The weight of implicit indicators is boolean. If patients ever took a kind of drug, the corresponding dimension would be marked as true. Otherwise, it would be marked as false. For example, there are *n* kinds of drugs in the dataset. Then the implicit indicators are generated as {*d*_1_,*d*_2_,…,*d*_*n*_}. If patient *i* ever used *d*_1_, the weight of *d*_1_ is 1. Otherwise, the weight of *d*_1_ is 0. This derived model is named ETSM-bool.
- **Times-Drug Method:** In this method, each dimension of the implicit indicator group also stands for a kind of drug. The weight of the implicit indicator group is the times that patients ever took a kind of drug. For example, there are *n* kinds of drugs in the dataset. The implicit indicators are also generated as {*d*_1_,*d*_2_,…,*d*_*n*_}. If patient *i* ever used *d*_1_ for three times, the weight of *d*_1_ is 3. This derived model is named ETSM-times.

#### Performance comparisons

Tables 5 and 6 provides the performance of the model with different input features in the experiment of predicting AKI 24 hours and 48 hours ahead. According to the table, the three models have their advantages and disadvantages. However, ETSM is able to utilize the drug combination information effectively.

### Feature importance analysis

In addition to validating our model’s predictive ability, we analyze the information provided by the trained model about feature importance. Based on the results, drug combinations indeed play a key role in determining AKI. Table 7 shows drug combinations with top 10 feature importance. Based on previous studies, most of the drugs listed in Table 7, such as norvancomycin, ibuprofen and naproxen, can cause drug-induced renal disorders individually or in combination[23, 24], which proves that the features selected by our model are reasonable. We hope that the drug combination we found can attract attention, provide suggestions for the study of inappropriate drug co-administration, and narrow the scope of clinical verification and testing.

**Table 7:** Drug combinations with top 10 feature importance

Rank	Drug Combination
1	5, 21
2	43, 69
3	21, 46
4	21, 43
5	21, 23, 43
6	1, 43, 69
7	5, 21, 50
8	21, 43, 69
9	21, 32
10	1, 21, 43

Note: Each number represents a kind of drugs. The number is the index for this drug in the ICUC dataset

1: norvancomycin

5: ciprofloxacin lactate and sodium chloride injection

21: indometacin enteric-coated tablets

23: piperacillin sodium/tazobactam sodium

32: ibuprofen

43: ceftazidime for injection

46: cefathiamidine for injection

50: aztreonam for injection

69: naproxen

## Discussion

In this paper, we propose an easy and straightforward time series model method to generate features from clinical data. In the experiment, our model provides a comparable result. In the internal validation, our model identifies AKI risky patients in the next 24 hours and 48 hours with an AUC of 0.81 and 0.78, respectively. In the external validation, it predicts AKI risky patients in the next 24 hours and 48 hours with an AUC of 0.95. Compared with state-of-the-art machine learning methods [7, 25, 26], our results are competitive. Such early prediction allows patients at high risk for AKI to obtain timely and early intervention and could mitigate patients’ morbidity and mortality. The data we used is readily available in the real clinical process.

Comparing to the recent predictive model based on the deep learning approach [10], our model focuses on exploring the effect of non deep learning methods on the AKI prediction task. In practical application, the deep learning model is a blackbox, and its interpretability is weaker than the ensemble tree model. Clinical data is a structured dataset, and each feature has a clear meaning. Deep learning models are hard to grasp the relationship between features and predicted values well. We have also noticed that there are many tools to help improve the interpretability of deep learning models, such as LIME (local interpretable model agnostic explanations), SHAP (Shapley additional plans), Captum and CD (contextual decomposition). These tools provide methods for visualizing the results and exploring the meaning of deep learning models. However, these methods are not perfect. Take LIME for an example, small perturbations that have minimal (or no) effect on the underlying model’s predictions, yet have significant effects on the explanations given be the interpreters meant to explain them [27]. Also, the training of SHAP is of exponential complexity for deep learning models, which is very time-consuming. But for tree algorithms, the training time complexity of SHAP can be optimized to be linear and the cost is greatly reduced [28]. The model’s interpretability decides whether it can provide valuable guidance for realistic events rather than just offering prediction results [29]. Important features selected by the interpretable model lead physicians to pay more attention to the key physiological indexes of patients. Such information is sometimes more meaningful than the predictive result since it is beneficial to offer patients appropriate medical interventions. Our creative feature generation method and XGBoost algorithm make ETSM well interpretable. For example, patients’ health condition is also under drug-induced risk in the practical clinical process. Some drugs may, individually or in combination, have the potential to trigger renal injury [30]. However, drug combination information is hard to catch through the usual feature generation methods. Unlike most existing prediction models that consider different drugs separately, we treat the drug combination as the implicit indicator group. Since the XGBoost algorithm that ETSM used is a collection of decision trees that are more interpretable than other classifiers, the decisions made by tree nodes are easily available and understandable. By combining the clinical meaning of implicit indicators, the drug-induced risk caused by drug combinations can be discovered through further analyzing the contribution of implicit indicators. In this way, our model could help lower drug-induced risk by offering physicians clinical medication guidance.

Moreover, aiming to further validate the contribution of medication information for AKI prediction, we carried out an ablation study where we removed the implicit indicator group. According to the experimental result, it is transparent that the performance declined when predicting AKI both 24 hours and 48 hours ahead. On the one hand, this experiment reflects that utilizing medication information effectively is quite beneficial to improve model performance. On the other hand, this ablation study has further verified that patients are indeed under drug-induced risk caused by drug co-administration, and implicit indicators are essential for AKI prediction.

As to imbalanced data, we found that the ratio of positive and negative samples would affect model performance. However, the better ratio setting of positive and negative class about model initialization needs further exploration. Therefore, we conducted a series of comparative experiments and selected the optimal ratio by evaluating model performance based on AUC, sensitivity, F1-score and AP comprehensively. Results show that when the ratio of positive to negative is 1:1 or 1:2, the model can get better performance. It should be noted that the ratio is also related to the dataset, but 1:1 or 1:2 is recommended.

At last, to further verify the rationality and superiority of our time series modeling method, we have tested two other feature generation methods ever used by previous researches, then validated the model performance. According to the results, our time series modeling method is overall better than several methods. However, Our methods can utilize drug combination information and help lower drug-induced risk. The experiment verified that our time series modeling method possesses high performance and practical clinical value.

Even better, our feature generation method is not confined to AKI prediction. The time series modeling method proposed in the paper is scalable and can be widely applied to other clinical prediction tasks.

## Conclusion

In this paper, we proposed a competitive prediction model for AKI based on an ensemble learning algorithm. Our model overcomes the difficulty caused by sparse and high-dimensional clinical data, providing comparable prediction results of AKI 24 hours and 48 hours ahead in both internal validation and external validation, which is paramount for ameliorating patients’ outcomes. Our model is quite competitive by comparison with other AKI prediction models, samely based on the machine learning method, predicting AKI nearly 2-day in advance. This time span between evidence of increased AKI risk and AKI onset is an ideal period for medical intervention. Additionally, we proposed a fast and straightforward time series modeling method for complex medication information and further verified that AKI patients are indeed under the drug-induced risk.

There are some limitations to this study. First, our model is developed from clinical data from ICU but is not implemented in other hospital departments, and its generalizability needs further validation. Second, the detailed information about the drug combination effect on AKI incidence needs further research.

In the future study, we will further explore the correlation between drug combination and AKI incidence.

## Data Availability

The ICUC dataset during the current study is available from the corresponding author on reasonable request. The MIMIC III dataset is downloaded from open-access datasets. The source code of ETSM is available on Github (https://github.com/echo0409/ETSM).

## Abbreviations

AKI: Acute kidney injury
ICU: Intensive care unit
ETSM: Ensemble time series model
TFIDF: Term frequency and inverse document frequency
IFICF: Indicator frequency and inverse cohort frequency
IF: Indicator frequency
ICF: Inverse corpus frequency
ICUC: ICU data in China
MIMIC: Medical information mart for intensive care
KDIGO: Kidney disease improving global outcomes
AUC: The area under the receiver operating curve
LIME: Local interpretable model agnostic explanations
SHAP: Shapley additional plans
CD: Contextual decomposition
